# Survival of a specialist natural enemy experiencing resource competition with an omnivorous predator when sharing the invasive prey *Tuta absoluta*

**DOI:** 10.1002/ece3.3396

**Published:** 2017-09-07

**Authors:** Anaïs Chailleux, Anthony Droui, Philippe Bearez, Nicolas Desneux

**Affiliations:** ^1^ Biopass ISRA‐UCAD‐IRD Dakar Senegal; ^2^ CIRAD UPR HortSys Montpellier France; ^3^ InVivo AgroSolutions Paris France; ^4^ INRA (French National Institute for Agricultural Research) Université Côte d’Azur CNRS UMR 1355‐7254 Institute Sophia Agrobiotech 06903 Sophia‐Antipolis France

**Keywords:** coexistence, functional trait, interspecific interaction, intraguild predation, *Macrolophus pygmaeus*, *Stenomesius japonicus*

## Abstract

Can specialist natural enemies persist in ecosystems when competing with omnivorous natural enemies for their shared prey? The consequences of omnivory have been studied theoretically, but empirical studies are still lacking. Omnivory is nevertheless common in nature and omnivorous predators coexist with specialists in many ecosystems, even when they are intraguild predators. This type of association is also common in agroecosystems in which biological control strategies are used. Our study provides an example of the outcome of such an association in the context of biological control of the invasive pest *Tuta absoluta* (Lepidoptera) in a tomato agroecosystem. The two natural enemies involved, that is, a specialist (*Stenomesius japonicus* (Hymenoptera) parasitoid) and an omnivore (*Macrolophus pygmaeus* (Hemiptera) predator), were able to coexist for 3 months in our experimental cages in the absence of metacommunity mechanisms (i.e., emigration and recolonization), contrary to theoretical expectations. However, they negatively affected each other's population dynamics. We found that spatial resource segregation was not a mechanism that promoted their coexistence. Regarding pest control, the specialist and omnivorous natural enemies were found to exhibit complementary functional traits, leading to the best control when together. Mechanisms that may have promoted the coexistence of the two species as well as consequences with regard to the inoculative biological control program are discussed.

## 1 INTRODUCTION

Multiple natural enemy species commonly attack single prey/host species (Hawkins, 1990; Hawkins & Mills, 1996; Polis, 1991; Polis & Strong, 1996; Price, 1971). How do they coexist in ecosystems when tapping a common resource is a key question to address in order to understand ecosystem functioning (Chase & Leibold, 2003; Finke & Snyder, 2008). It has been established that relatively strong intraspecific competition combined with relatively weak interspecific competition can foster species coexistence and promote biodiversity (e.g., Chase, 2003; Hutchinson, 1959; MacArthur, 1970; Mc Kane et al., 2002).

Four mechanisms are recognized as being effective in decreasing interspecific competition: (i) aggregation behavior (Hanski, 1981; Ives, 1988), (ii) diet breadth (Bonsall & Wright, 2012; Itino, 1992; Miller, 1967; Peers, Thornton, & Murray, 2012), (iii) resource segregation (Bonsall, Hassell, & Asefa, 2002; MacArthur, 1972), and (iv) trade‐offs in life history traits (Bonsall et al., 2002).

Stable associations between species sharing the same resources and having different diet breadths are common in nature (Coll & Guershon, 2002; Polis, Myers, & Holt, 1989), but such associations are puzzling for ecologists because of the dearth of relevant theoretical explanations (Krivan and Diehl 2005). Empirical experiments are lacking, especially regarding studies on the effects of true omnivory, that is, feeding on different trophic levels, such as on plants and on herbivores, and not only on different herbivore prey species (Coll & Guershon, 2002; Pimm & Lawton, 1978).

Old theories have indicated that, at equilibrium, the presence of an omnivorous species can destabilize food webs (Coll & Izraylevich, 1997; Pimm & Lawton, 1978), and as such, omnivory ought to be uncommon in real situations. These theories were then revised to suggest that omnivory could actually stabilize ecosystems (Lalonde, McGregor, & Gillespie, 1999; Mc Cann & Hastings 1997). This last hypothesis hints that switching from one trophic level feeding to another trophic level feeding decreases the impact on food species when it is at low densities. Another factor that may stabilize ecosystems is the relatively usual poor searching efficiency of omnvorous species (Eubanks & Styrsky, 2005; Lalonde et al., 1999; Peers et al., 2012). It is likely that omnivory, by decreasing predator pressure on the shared prey, may also limit the strength of the competition between the omnivorous predator and other natural enemies within the same guild. There are potentially two main limits in these theories. First, omnivorous diet breadth enables predators to persist at low prey levels by feeding on plants (Crawley, 1975; Eubanks & Denno, 1999; Pimm & Lawton, 1977, 1978; Walde, 1994). They can thus persist at a reduced prey density at which more specialized competitors would not be able to survive. Second, omnivorous predators are often involved in intraguild predation, that is, when one of the competitors attacks and feeds directly on the other one (Polis et al., 1989). The two competitors interact directly, reducing the potential persistence of the specialist that is the focus of predation from the omnivorous competitor.

Omnivorous predators are frequently used in combination with specialist natural enemies to protect crops from insect pests in agroecosystems (see Snyder & Ives, 2003). Many species of biological control agents are also known to be intraguild predators and to also feed on plants, that is, true omnivory (Pimm & Lawton, 1978). For example, spiders are known to feed on pollen and to attack other predators (Cohen, 1998; Hodge, 1999; Peterson, Romero, & Harwood, 2010; Smith & Mommsen, 1984). Moreover, some predatory bugs, such as Heteropterans, are known to feed on sap or pollen while also feeding on various predators (Schmidt et al. 1998, Vandekerkhove & De Clercq, 2010; Biondi et al., 2016; Perdikis & Arvaniti, 2016). These predators, having access to multiple food resources, are thus regularly considered as superior competitors that may exclude specialist feeders at the population level (Grover, 1997).

We ask whether omnivory allows the specialist natural enemy to survive in a closed system, for example, a glasshouse environment, where there are no metacommunity mechanisms (i.e., emigration and recolonization) at the landscape level? Most experimental studies on predation/parasitism have only examined the effects of one natural enemy species at a time (Sih, Englund, & Wooster, 1998) or of multiple natural enemies with the same diet breadth, for example, between specialists or between omnivores (Batchelor, Hardy, Barrera, & Pérez‐Lachaud, 2005; Moreno‐Ripoll, Agusti, Berruezo, & Gabarra, 2012; Sanders, Schaefer, Platner, & Griffi, 2011). Few field studies have addressed issues pertaining to interspecific competition between specialist and omnivorous species and the impact on natural enemies‐prey/host dynamics (Godfray, Hassell, & Holt, 1994).

In this study, we assessed competitive interactions between two natural enemy species—a specialist parasitoid and an omnivorous predator. Both of them attack *Tuta absoluta* Meyrick (Lepidoptera: Gelechiidae), a major tomato invasive pest (Biondi, Guedes, Wan, & Desneux, 2018; Campos, Adiga, Guedes, Biondi, & Desneux, 2017; Desneux, Luna, Guillemaud, & Urbaneja, 2011; Desneux et al., 2010; Sylla et al., 2017). The omnivorous species was the predatory bug *Macrolophus pygmaeus* Rambur (Hemiptera: Miridae). It is mainly used to control whiteflies and is able to feed on plant food sources such as sap or pollen (Bompard, Jaworski, Bearez, & Desneux, 2013; Calvo, Blockmans, Stansly, & Urbaneja, 2009; Jaworski, Chailleux, Bearez, & Desneux, 2015). This predator was recently used in *T. absoluta* control programs and preferentially attacks eggs and rarely young larval instars of the pest (Urbaneja, Monton, & Molla, 2009). At the same time, several ecto‐ or endoparasitoids (mainly Eulophidae, Braconidae, and Ichneumonidae) have also been reported to attack *T. absoluta* in the Mediterranean Basin (see e.g., Zappalà et al., 2013). Among them, the idiobiont ectoparasitoid *Stenomesius japonicus* Ashamed (Hymenoptera: Eulophidae), which naturally occurs in newly invaded areas (Zappalà et al., 2013), preferentially attacks old larvae (3rd instar larvae; Chailleux, Desneux, Arnó, & Gabarra, 2014). It should be possible to use this parasitoid species through inoculative releases to control *T*. *absoluta*, although its capacity to persist in tomato crops in the presence of *M. pygmaeus* remains to be assessed. Moreover, mirid predators can exert kleptoparasitism and/or intraguild predation on *T. absoluta* larval parasitoids by feeding on paralyzed and parasitized host larvae or by directly attacking the juvenile parasitoids developing on the host larvae (Chailleux, Wajnberg, Zhou, Amiens‐Desneux, & Desneux, 2014; Naselli et al., 2017). The objectives of this study were (i) to quantify the outcome of a frequent species association with an ecosystem (i.e., a specialist natural enemy and an omnivorous natural enemy), both feeding on the same prey, and (ii) to identify resource utilization patterns that could promote the coexistence of the two natural enemies.

## 2 MATERIALS AND METHODS

### 2.1 Biological materials

Five‐week and six‐week‐old pesticide‐free tomato plants, *Solanum lycopersicum* L. (cv. Betalux), were used in the laboratory and glasshouse experiments, respectively. They were grown in climatic chambers (24 ± 1°C, HR: 65%, photoperiod 16L:8D) with a daily‐applied nutrient solution, and no pesticide was used. Insects were reared in growth chambers (25 ± 1°C, RH 70 ± 10%, 16L:8D). A colony of the shared prey, that is, the leafminer *T. absoluta,* was set up using glasshouse‐collected individuals in July 2009 at INRA, Alenya, France (initial number of individuals = 190). The colony was kept in plastic cages (55 × 75 × 80 cm width:height:depth), containing tomato plants for leaf‐mining larvae feeding, and honey was provided for imago feeding. The *Stenomesius japonicus* colony was reared in cages (same as for *T. absoluta* rearing) with a constant supply of tomato infested with *T. absoluta* larvae, and honey droplets were provided on the plants as food for imagoes. The laboratory rearing was initiated using a mix of individuals from Spain and France (*n *=* *11, *n* = 7, respectively) collected on commercial tomato crops. The predator species, *M. pygmaeus*, originally came from the Biotop commercial insectary (France). They were then reared for at least one generation on tobacco and fed on *Ephestia kuehniella* UV‐sterilized eggs in cages under the same environmental conditions as described above.

### 2.2 Population dynamics experiment

#### 2.2.1 Experimental setup

The experiments were carried out in cages (70 × 100 × 100 cm width:height:depth) covered with insect‐proof mesh and placed in a glasshouse in the facilities of the INRA AgroBiotech Institute (Sophia Antipolis, France). Six 2‐l pots, each containing a tomato plant (seven to eight fully developed leaves), were placed inside each cage. Tomato plants were woven vertically on stakes, and side stems were removed every week and left underneath the plants to allow insect eggs to hatch. Plants were watered automatically with a nutrient solution, and pesticide applications were strictly avoided. The temperature and humidity were regulated with fog, shade, and airing and kept as close as possible to 25°C (range 9.8–39.5°C); RH: 67.5% (range 19.0%–95%) under natural ambient light (May–July 2012).

The following three combinations of natural enemies were compared: (i) *T. absoluta* + *S. japonicus,* (ii) *T. absoluta* *+ M. pygmaeus,* and (ii) *T. absoluta* + *S. japonicus* + *M. pygmaeus*. These three combinations were tested on two *T. absoluta* densities independently to test for potential effects of prey density on the population dynamics obtained. The low density corresponded to four pairs (one male and one female, hereafter called pair) and the high density to 16 pairs of young imagos (<1 week old) released per cage at the beginning of the experiment. The two natural enemy individuals were released at a ratio of two pairs per plant, with a total of 12 pairs per cage and per species. Consequently, the treatment with the two natural enemy species contained twice as many natural enemies as those with only one species. Treatments with only one natural enemy were repeated three times, while the one with both natural enemies was repeated four times. As two pest densities were tested for each natural enemy treatment, in total, 20 cages were established. Treatments were spatially randomized within the glasshouse.

**Figure 1:**
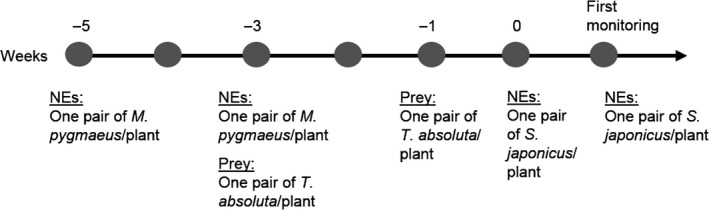
Diagram of the release strategy used for the cage experiment until the first recording. Each insect species (natural enemies (NEs) and prey) was released two times

Each species was released twice (i.e., half‐quantities each time) (Figure 1): a first release and then a second one after a time interval corresponding to the half of their respective life‐cycle duration (i.e., 1 week for *S. japonicus*, and 2 weeks for *T. absoluta* and *M. pygmaeus*). Young *M*.* pygmaeus* adults (<3 days) were released first, that is, 2 weeks before the first *T. absoluta* release. Commercially available UV‐sterilized eggs of *E. kuehniella* (Biotop, France) were placed on the plant as an initial food source for *M. pygmaeus* to ensure its establishment on the crop. Preventive inoculative releases (i.e., release and eventually feeding of predators early in the crop season before any pest infestation) are recommended by the company selling the predator. *Stenomesius japonicus* imagos (mixed ages) were released once larvae of *T. absoluta* had reached the ideal stage (3rd instar larvae) for parasitoid offspring production (Chailleux, Desneux et al., 2014). Insect releases began on the 30 April 2012, and the last release of *S. japonicus* was done on 11 June 2012. Monitoring started at week 1, on 13 June, that is, 1.5 months after the first release and the same week as the last parasitoid release.

#### 2.2.2 Monitoring and sampling

In each cage, two plants were selected every week for monitoring. Plants were monitored weekly for 8 weeks after the last insect releases, so each plant was monitored twice. *M. pygmaeus* adults and nymphs were counted on the entire selected plants. *T. absoluta* eggs and larvae were monitored on six leaves, two leaves were selected at random from the upper, middle, and bottom third of each selected plant. *T. absoluta* larvae were observed by shining a torch lamp under each leaf, and eggs were observed with a hand lens. All *S. japonicus* adults observed in the cages (on all the plants and on the cage walls) were counted. Parasitism was evaluated on six leaflets (containing *T. absoluta* mines) per selected plant. Leaflets were collected, and for each leaflet, one mine containing a *T. absoluta* larva was dissected subsequently in the laboratory under a binocular microscope to count the number of *S. japonicus* larvae, eggs, and pupae. Hence, a total of 12 mines containing a *T. absoluta* larva were examined weekly per cage. After observation, the leaves were put back in their original cage.

### 2.3 Within‐plant resource partitioning experiment

The impact of resource partitioning along the main vertical shoot axis of the tomato plant on the strength of resource competition was tested under laboratory conditions. Forty‐five potted tomato plants were covered with a plastic cylinder (15 × 30 cm diameter:height) closed on the top with a mesh. Seventeen *T. absoluta* eggs and 10 larvae were deposited on each plant with a paint brush. The three following treatments were set up and tested: (i) eggs on the upper third of the plant and larvae on the bottom third of the plant (i.e., pest instar distribution commonly observed within tomato plants, Torres, Faria, Evangelista, & Pratissoli, 2001); (ii) the reversed distribution (i.e., eggs on the bottom third and larvae on upper third); and (iii) mixed eggs and larvae all over the plant. The *T. absoluta* eggs used were 0‐to‐12 hr old, and the larvae were late second and early third instars. Five *S. japonicus* and one *M. pygmaeus* females were then introduced in the cylinder 1 hr after the *T*. *absoluta* larvae to allow the larvae to dig mines. Three days later, natural enemies were removed, and mines were collected to assess, under a binocular microscope, egg predation, and larva parasitism and predation. Fifteen replications, that is, 15 plants, were carried out per treatment.

### 2.4 Statistical analyses

In the population dynamics experiment, differences in population dynamics of pests and natural enemies among treatments were analyzed using generalized estimating equations (GEE) with autoregressive correlation structure to treat repeated measures over time. A GEE based on Poisson‐distributed data with a log‐link function was applied for the numbers of *T. absoluta*,* M. pygmaeus,* and *S. japonicus* imagos. A binomial distribution was used for the parasitism rate using the nontransformed numbers of parasitized and nonparasitized larvae recorded. For the *T. absoluta* larva and egg dynamics, the factors tested were the natural enemy combination, the initial *T. absoluta* release quantity (i.e., pest density factor), and the date corresponding to the time factor. The number of *T. absoluta* parasitized larvae per leaf was calculated as follows: ([number of *T. absoluta* parasitized larvae found in laboratory opened mines]/[total number of *T. absoluta* larvae observed in laboratory opened mines] × [number of *T. absoluta* larvae counted on the six leaves recorded])/6. For the *S. japonicus* imago and the parasitism dynamics, the factors tested were the predator presence, the initial *T. absoluta* release quantity, and date. Finally, for the predator population dynamics, the factors tested were the parasitoid presence, the initial *T. absoluta* release quantity, and date. In all cases, interactions between factors were tested, but they are only presented in the Results section when statistical significance and interest for the study are essential.

The resource partitioning experiment results were analyzed using a GLM for Poisson data with the treatments as a factor. When necessary, means were separated using a least significant difference post hoc test (LSD test) for multiple comparisons. All statistical analyses were performed using R software (R Development Core Team 2009) with the *stats* package (for fitting GLM models), the *geepack* package (for fitting GEE models), and the *multcomp* package for post hoc tests.

## 3 RESULTS

### 3.1 Population dynamics experiment

**Figure 2:**
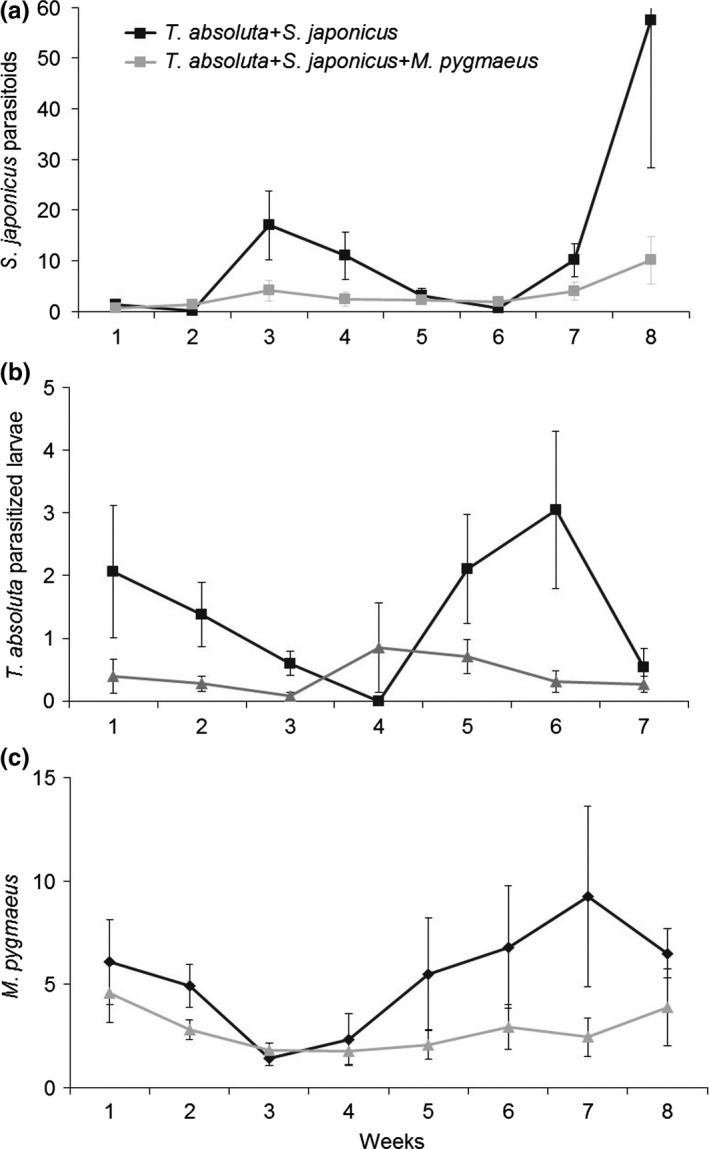
Mean (±*SE*) number of (a) larval parasitoid (*Stenomesius japonicus*) adults per cages, (b) leafminer (*Tuta absoluta*) parasitized larvae per leaf, (c) omnivorous predators (*Macrolophus pygmaeus*) per plant, over 8 weeks when alone with the shared leafminer prey (*T. absoluta*), or with the concomitant presence of the competitor species. This graph represents pooled data for both *T. absoluta*‐tested densities for each treatment. The last error bar was truncated to preserve the graph readability

**Figure 3:**
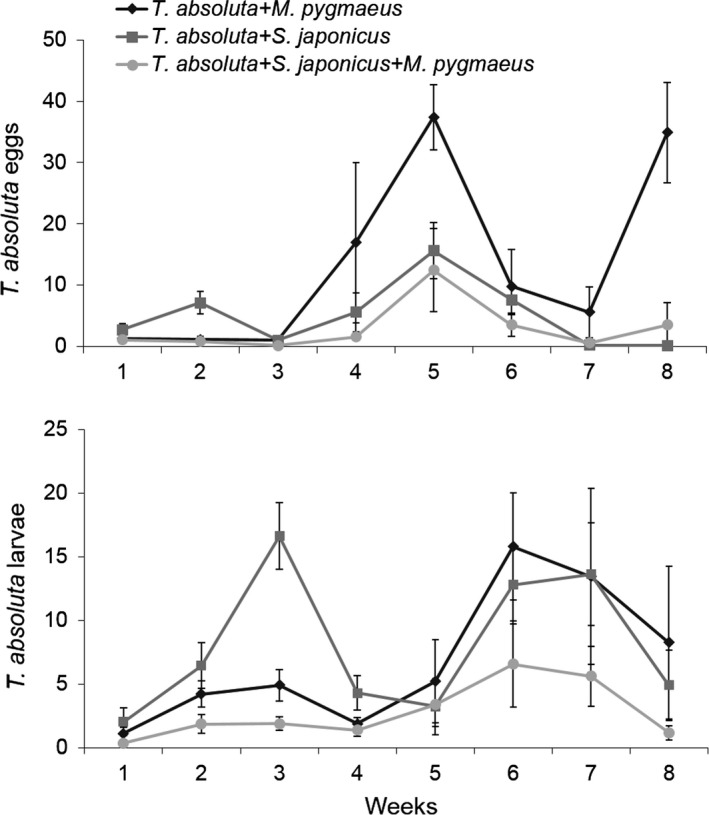
Mean (±*SE*) number of the leafminer *T. absoluta* eggs (top) and larvae (bottom) per leaf over 8 weeks in the presence (i) of the larval parasitoid *Stenomesius japonicus*, (ii) of the predator *Macrolophus pygmaeus,* and (ii) of *S. japonicus + M. pygmaeus*. This graph represents pooled data for both *T. absoluta*‐tested densities for each treatment

All species were observed at the end of the experiment in the cages from which they were initially released. No local extinctions were noted with regard to the natural enemies or the host/prey, indicating that natural enemies might coexist, at least over the length of the experiment. The results of the two *T. absoluta* density tests were pooled in the figure presenting the insect population dynamics (Figures 2 and 3) because no significant density effects were observed. The number of *S. japonicus* adults in the cages (Figure 2) was significantly affected by both the predator presence and the date (χ² = 13.03, *df* = 1, *p* < .001; χ² = 82.93, *df* = 7, *p *< .001, respectively). A most significant increase in parasitoid number was observed on week 8, especially in the treatment without predators (Figure 2). However, there was no effect of *T. absoluta* release density on the parasitoid population levels (χ² = 0.36, *df* = 1, *p *= .548).

A significant effect of the predator presence on the parasitism rate of *T*. *absoluta* larvae by *S. japonicus* was observed only in interaction with the week and density (predator:week: χ² = 80.03 *df* = 6, *p* < .001 and predator:density: χ² = 4.60, *df* = 1, *p* < .032) (Figure 2). The effect of the week factor alone was significant (χ² = 224.90, *df* = 6, *p *< .001) (Figure 2). Finally, the *T. absoluta* release density also had a significant effect on the parasitism rate (χ² = 1.68e + 08, *df* = 1, *p* ≤ .001).

Predator numbers were significantly affected by the presence of the larval parasitoid (χ² = 4.33, *df* = 1, *p *= .037) and by the date (χ² = 53.92, *df* = 7, *p* < .001) (Figure 2); the mirid population was higher at the end of the experiment and in the absence of the parasitoid. The *T. absoluta* release density had no effect on their population (χ² = 0.003, *df* = 1, *p *= .955).

*Tuta absoluta* eggs and larvae (Figure 3) were significantly affected by the natural enemy combination used (χ² = 14.72, *df* = 2, *p *< .001; χ² = 10.19, *df* = 2, *p* = .006, for eggs and larvae, respectively) and by the date (χ² = 494.92, *df* = 7, *p* < .001; χ² = 94.31, *df* = 7, *p* < .001, for eggs and larvae, respectively). The *T. absoluta* release density had no effect on the egg number (χ² = 0.24., *df* = 1, *p* = .621) and had a marginally significant effect on the larva number (χ² = 2.92, *df* = 1, *p *= .088). Overall, fewer *T. absoluta* eggs and larvae were recorded in the treatment in which both natural enemies were released (Figure 3).

### 3.2 Within‐plant resource partitioning experiment

**Figure 4:**
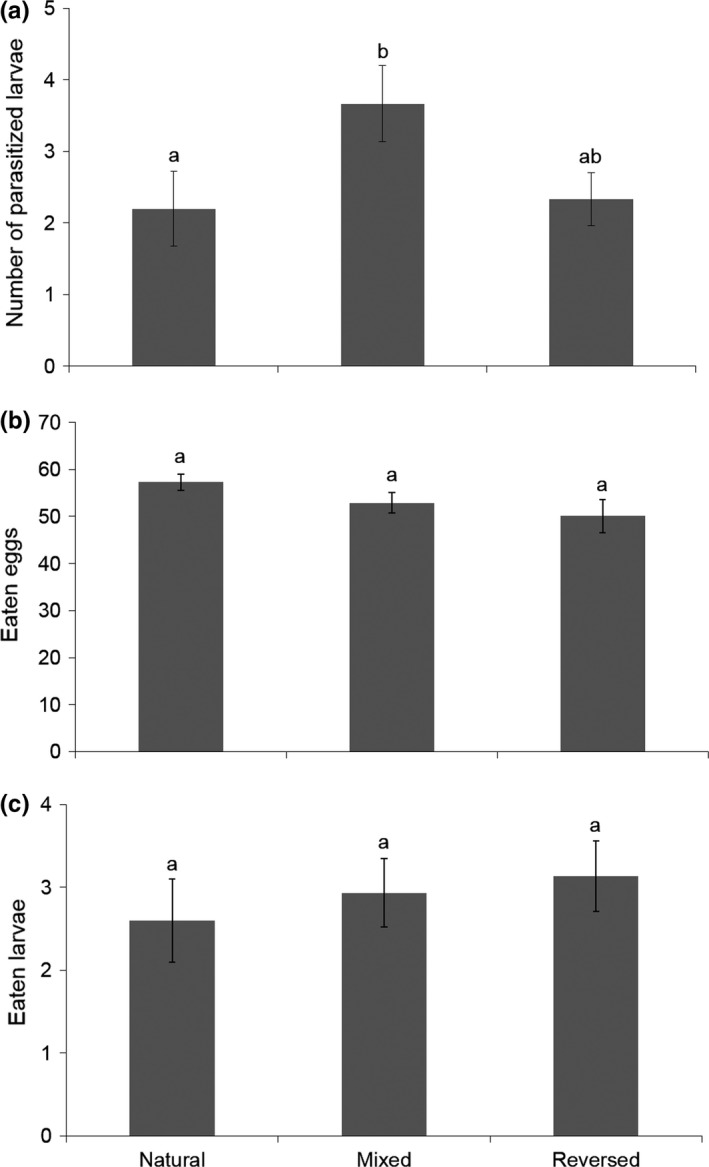
Mean (±*SE*) number of (a) the leafminer *Tuta absoluta* larvae parasitized by the larval parasitoid *Stenomeisus japonicus*, (b) the leafminer *T. absoluta* eggs, and (c) larvae eaten by the omnivorous predator *Macrolophus pygmaeus* in the concomitant presence of the competitor species after 3 days under laboratory conditions in three *T. absoluta* stage distributions along the plant axis: (i) when *T. absoluta* eggs were on the upper part of the plant and larvae on bottom part of the plant (i.e., natural), (ii) when *T. absoluta* eggs and larvae were mixed all along the plant axis (i.e., mixed), or (ii) when *T. absoluta* eggs are on the bottom part of the plant and larvae on upper part of the plant (i.e., reversed). Columns bearing the same letter are not significantly different at *p* < .05

Although the number of larvae parasitized by *S. japonicas* was marginally significantly affected by the resource distribution (*F*_2,42_ = 2.82, *p* = .071), the highest level of parasitism was obtained for the treatment in which eggs and larvae were mixed along the plant axis (Fisher's LSD post hoc test: mixed vs. reversed: *Z* = 1.89, *p *= .059; and natural vs. mixed *Z* = −2.09, *p* = .036) (Figure 4). The “natural” and “reversed” treatments were not statistically different (Fisher's LSD post hoc test: *Z* = −0.22 and *p* = .827). The numbers of eggs and larvae consumed by *M. pygmaeus* were not affected by the resource distribution (*F*_2,34_ = 2.43, *p* = .103; *F*_2,42_ = 0.38, *p* = .684, for eggs and larvae, respectively; Figure 4).

## 4 DISCUSSION

The results showed that coexistence was possible between the two natural enemy species, despite the asymmetry in their diet breadth and the occurrence of kleptoparasitism (Chailleux, Wajnberg et al., 2014): *S. japonicus* and *M. pygmaeus* became successfully established in all treatments in which they were released and were present throughout the duration of the experiment. Nevertheless, for both biocontrol agent species, their population sizes were significantly lower when they were in competition with each other versus when they were alone. The lowest population of the shared host/prey was observed when the two natural enemies were jointly present.

Contrary to the results of Bogran, Heinz, and Ciomperlik (2002), we found no or marginal effects of the *T. absoluta* initial release quantity on the population dynamics observed. In our experiment, the omnivorous predator was released before the pest, which makes sense from an ecological point of view as the predator is able to feed on the plant. This also mimics preventive releases of omnivorous predators, as usually done by tomato producers (Calvo, Lorente, Stansly, & Belda, 2012). Moreover, *M. pygmaeus*, as other heteropteran predators (Salehi, Fatemeh, Arash, & Zandi, 2016), has a functional response when fed with *T. absoluta* eggs or larvae, that is, attacking more prey when more prey are available (Jaworski, Bompard, Genies, Amiens‐Desneux, & Desneux,^2013^). This response may have led to an initial reduction in pest egg number in proportion to the initial pest release density. Indeed, *M. pygmeus* was released first in the cages and could have consumed more eggs in the high *T. absoluta* density cages than in the low ones, thus equalizing the two initial densities to almost similar levels.

The successful coexistence of the two competitors during the experiment suggests that some mechanisms probably decreased the strength of the competition (either exploitative competition of the shared resources or kleptoparasitism), thus avoiding the parasitoid exclusion we expected (Pimm & Lawton, 1978; Krivan and Diehl 2005). Lalonde et al. (1999) suggested that poor prey foraging efficiency for an omnivorous predator reduces its impact on herbivore populations. Indeed, it is known that *M. pygmaeus* exhibits a low level of predation and spends a short time foraging for prey, mainly because the females spend most of their time on the stem in search of suitable oviposition sites (Montserrat, Albajes, & Castañé, 2004). However, prey is usually found on the leaves but seldom on the stem, so *M. pygmaeus* females may seldom encounter prey. Such low efficiency in resource use may account for the coexistence of the two natural enemy species by reducing both exploitative competition and kleptoparasitism. Differences in resource utilization have been theoretically shown to promote coexistence between species (Wilson, Osenberg, Schmitt, & Nisbet, 1999). In practice, Brown, Kotler, and Mitchell (1997) demonstrated the ability of a forager to profitably harvest resources at low abundances, thus allowing them to utilize the resources left behind by the less efficient forager.

A second mechanism may reduce the impact of kleptoparasitism. We initially hypothesized that one of the mechanisms potentially reducing the strength of kleptoparasitism could be resource segregation along the plant axis. If the predator feeds preferentially on eggs, as demonstrated by Urbaneja et al. (2009), it may stay most of the time where eggs are the most abundant, that is, on upper part of the plant, thus reducing the probability of encountering larvae attacked by the parasitoid species, as larvae are instead generally located on the middle part of the plant (Torres et al. 2001). However, our laboratory experiments did not provide any evidence of such a mechanism and did not indicate that resource segregation along the main vertical plant axis was the mechanism reducing kleptoparasitism. We cannot exclude the possibility that using taller plants might have led to different results. Nevertheless, segregation was artificially amplified in our experimental setup, and this could have counterbalanced the relatively small plant size.

Our results tended to demonstrate that the predator also suffered from the parasitoid presence. This may be a consequence of the fact that the parasitoid markedly decreased the density of the shared resource. Specialists are generally considered to have a higher effect on herbivore populations and to respond better to herbivore population fluctuations than generalists (Snyder & Ives, 2003). Moreover, in our study, the parasitoid species may have had a greater effect on the pest population than the predator because parasitoid females attack later development stages of the pest species that are more likely to reach the reproductive stages successfully, while a portion of eggs killed by the predator may also have died because of natural mortality or climate (Miranda, Picanco, Zanuncio, & Guedes, 1998). Similar hypotheses have also been proposed to explain the superiority of a parasitoid that prefers bigger hosts, thus removing individuals with a higher reproductive value (Lin & Ives, 2003).

In terms of pest biological control, the pest population dynamics obtained with each of the two natural enemies present differed. As a specialist, *S. japonicus* exerted less efficient control of *T. absoluta* growth just after release, with stronger suppression noted thereafter. In contrast, the omnivorous predator caused an immediate decrease in the pest population growth rate but provided poor control later on. The short life cycle and specificity of parasitoids can allow them to mount a strong numerical response when prey outbreaks occur, perhaps leading to outbreak suppression (Berryman, 1992; Hassell, 1980; Hassell & May, 1986; Murdoch, 1994; Turchin, Taylor, & Reeve, 1999). On the contrary, omnivorous predators have a longer generation time than herbivores. Hence, even if there is a numerical response to changes in the density of a single herbivore species (e.g., Symondson, Sunderland, & Greenstone, 2002), the response is unlikely to occur quickly enough to lead to outbreak suppression (Debach & Rosen, 1991; Hassell & May, 1986). However, their ability to appear in the crop before the pest can ensure immediate control (Calvo et al., 2012; Snyder & Ives, 2003). When both the parasitoid and the omnivorous predator were present, *T. absoluta* dynamics changed according to the impacts of both natural enemies, that is, the initial pest population increase was as low as the treatment with only the predator, whereas the pest densities peaked at levels similar to that of the treatment with the parasitoid alone. This study corroborated the findings of other experimental studies in support of the idea that intraguild predation, even when reducing the intraguild prey population, does not hamper the pest control efficiency (e.g., Bilu & Coll, 2007; Heinz & Nelson, 1996; Messelink, Bloemhard, Sabelis, & Janssen, 2013; Snyder & Ives, 2003). In our experiment, when the two natural enemy species were together, there was twice as many as natural enemies as in the treatment with only one species. An additive effect of their two efficiencies explains the lowest level of the shared prey in this treatment and reveals that (i) the negative interactions between the two natural enemy species did not cancel the interest of having them together instead of choosing only one and (ii) the efficiency of one of the two natural enemies did not hide the efficiency of the other one, contrary to the results of Calvo, Soriano, Stansly, and Belda (2016) who tested different natural enemy species. Therefore, from a practical standpoint, our results suggested that efficient biological control programs could be based on joint inoculative releases of an omnivorous and a specialist natural enemy that appeared to have complementary functional traits. We also obtained promising results regarding the development of biological control programs against *T. absoluta* that rely on endemic biocontrol agents.

The present study provides an example of the outcome of a four level food web associating a specialist natural enemy and an omnivorous one when various parameters regulating population dynamics occur simultaneously, but in the absence of metacommunity mechanisms (i.e., emigration and recolonization). We showed that a specialist (here a parasitoid), affected by both exploitative competition and kleptoparasitism, was able to survive in the presence of the omnivorous predator, and was able to reduce the population density of this latter predator. Thus, in our biological model and experimental conditions, omnivory did not lead to specialist exclusion and parameters favoring coexistence—not resource segregation but possibly differences in foraging efficiency—seemed to outweigh the omnivory negative effects on coexistence.

## AUTHORS’ CONTRIBUTIONS

AC designed the experiments, helped to carry out the experiments, did the statistical analyses, and wrote the article. AD led the experiments and contributed to the statistical analyses. PB produced the biological material and helped to carry out the experiments. ND is the head of the laboratory and he contributed to the experiment design and to the article writing.
